# Investigating Causal Relations Between Sleep-Related Traits and Risk of Type 2 Diabetes Mellitus: A Mendelian Randomization Study

**DOI:** 10.3389/fgene.2020.607865

**Published:** 2020-12-15

**Authors:** Xue Gao, Heli Sun, Yu Zhang, Long Liu, Juping Wang, Tong Wang

**Affiliations:** ^1^Department of Health Statistics, School of Public Health, Shanxi Medical University, Taiyuan, China; ^2^Department of Mathematics, School of Basic Medical Sciences, Shanxi Medical University, Taiyuan, China

**Keywords:** insomnia, sleep duration, morningness, type 2 diabetes mellitus, causal relations, Mendelian randomization

## Abstract

**Objective:**

Extensive literature put forward the link between sleep and type 2 diabetes mellitus (T2DM), however, little is known about the underlying causality of the associations. Here we aim to assess the causal relationships between five major sleep-related traits and T2DM.

**Design, Setting, and Participants:**

Two-sample Mendelian randomization (MR) was utilized to investigate the potential causal relations. Independent genetic variants associated with five sleep-related phenotypes—insomnia, sleep duration, short sleep duration, long sleep duration, and morningness—were chosen as instrumental variables to estimate the causal associations with T2DM. Summary statistics were acquired from the genome-wide association studies of UK Biobank and 23andMe (for sleep-related measures), the DIAbetes Genetics Replication And Meta-analysis and the FinnGen (for T2DM).

**Main Methods:**

Individual Cochran’s Q statistic was applied to remove the pleiotropic instruments, global Q statistics and MR-Egger regression were adopted to test for the global heterogeneity and horizontal pleiotropy of the screened instruments, respectively. Two T2DM cohorts were selected to analyze their associations with sleep traits. A modified inverse variance weighted (IVW) estimate was performed to combine the ratio estimators from each instrument and acquire the causal estimate, alternative methods including IVW with first-order weights, simple and weighted median estimations, and MR-Egger regression were conducted as sensitivity analyses, to ensure the robustness and solidity of the findings.

**Results:**

Two-sample MR supported findings for an adverse effect of genetically predicted insomnia on T2DM risk (odds ratio [OR] = 1.14, 95% confidence interval [CI]: 1.09–1.19, *p* = 1.29E–08) at the Bonferroni-adjusted level of significance (*p* < 0.005). We further investigated the causal role of T2DM on insomnia but obtained a non-significant estimation. There was also little evidence for the causal effect of other sleep-related measures on T2DM. Results were largely consistent when leveraging two different T2DM cohorts, and were robust among various sensitivity analyses.

**Conclusion:**

Findings provide significant evidence for an adverse effect of insomnia on T2DM risk. The study extends fundamental knowledge to further understanding of the pathophysiological mechanisms of T2DM, and points out the non-negligible role of insomnia on epidemiologic intervention and clinical therapeutics of T2DM.

## Introduction

Type 2 diabetes mellitus (T2DM), is a chronic condition that describes a group of metabolic disorders characterized by insulin resistance (Arnold et al., 2018). The global prevalence of T2DM was estimated to be around 450 million. If current trends continue, the cases will rise to 700 million by 2045 (Saeedi et al., 2019). In the past three decades, the prevalence of T2DM has risen doubled worldwide, leading to a heavy health burden of disability and mortality (Roglic et al., 2005).

Quantity and quality of sleep are considerable lifestyle factors that influence the development of T2DM. Several studies have reported the associations of sleep-related traits with T2DM (Kawakami et al., 2004; Meisinger et al., 2005). One retrospective cohort study indicated that insomnia imparts an increased risk of T2DM (LeBlanc et al., 2018). Moreover, a systematic review of prospective studies showed a U-shaped association between sleep duration and the risk of T2DM (Brady et al., 2018). Consistent with this, a cross-sectional study demonstrated that both short and long sleep duration are associated with an increased risk of T2DM (Chaput et al., 2007). Besides, sleep chronotype may also play an important role in the risk of T2DM. One study found that morningness was associated with lower HbA1c, which induced a high risk of T2DM (Iwasaki et al., 2013).

Despite previous observational evidence of the relationship between sleep and T2DM, there are also inconsistent results (Björkelund et al., 2005; Lai et al., 2013). Also, several studies showed that T2DM may also lead to sleep disorders, it is not certain whether sleep causally influences the risk for T2DM, or T2DM reversely affects sleep (Hein et al., 2018; Dong et al., 2020). Moreover, observational studies are open to confounding, which can hardly be ruled out. Therefore the causal relationship between sleep and T2DM remains unclear.

Mendelian randomization (MR) can offer essential evidence for the causal inference (Emdin et al., 2017). Commonly, MR utilizes genetic variants associated with the exposure but does not directly affect the disease outcome as instrumental variables. The alleles of the variants are distributed randomly at conception, thus the predisposition for the exposure that is proxy by the genetic variants is distributed randomly, in this way the MR framework can approximate the RCT design. Besides, since the formation of the genetic variants is stable from conception on, they always precede the development of disease outcomes and other possibly confounding factors. Based on the distinct nature of genetic variants, MR can overcome the downsides of traditional observational studies such as unmeasured confounding and reverse causation, and provide a more valid estimation for the causal relationship between the exposure and the outcome (Agoritsas et al., 2017). Benefiting from the Genome-wide association studies (GWAS) which unraveled the association of genetic variants with phenotypes, the summarized data-based MR methods promote the causal inference of various traits with diseases considerably, and are prevailingly suggested and extensively applied in recent studies (Gao et al., 2019; Porcu et al., 2019; Sun et al., 2020).

There have been some studies exploring the causality between sleep and T2DM based on MR design. For example, Wang et al. (2019) examined the relationship of sleep duration with the risk of diabetes but found that they were not causally related. Bos et al. (2019) investigated the effect of total, short and long sleep duration on glycemic traits but the results provided little evidence for the causal role. Despite this, a comprehensive causal relationship of different sleep measures with T2DM has not been identified. As sleep is potentially modifiable, identifying the causal association of sleep and T2DM has substantial implications for preventing T2DM and improving population health. Herein, in the current study, we utilized the MR framework to investigate the causal relations between a wide range of sleep-related phenotypes (including insomnia, sleep duration and morningness) and T2DM.

## Materials and Methods

### Study Sample and Data Sources

A total of five cohorts (three for sleep-related traits and two for T2DM) were included in our study. Summary-level data had been made publicly available, and ethical approval had been obtained in the original studies (Table 1).

**TABLE 1:** GWAS cohorts used in this study.

Phenotype	First author (year)	Sample size	Consortium	PubMed ID
Insomnia	Jansen et al. (2019)	397,959 cases, 933,051 controls	UKB/23andMe	30804565
Sleep duration	Dashti et al. (2019)	446,118	UKB	30846698
Short sleep duration	Dashti et al. (2019)	106,192 cases, 305742 controls	UKB	30846698
Long sleep duration	Dashti et al. (2019)	34,184 cases, 305742 controls	UKB	30846698
Morningness	Jones et al. (2019)	372,765 cases, 278,530 controls	UKB/23andMe	30696823
T2DM	Scott et al. (2017)	26,676 cases, 132,532 controls	DIAGRAM	28566273
T2DM	2020	17,616 cases, 114,000 controls	FinnGen	NA

*T2DM, Type 2 diabetes mellitus; UKB, UK Biobank; DIAGRAM, DIAbetes Genetics Replication And Meta-analysis.*

Summary-level data for insomnia were derived from the largest available meta-analysis of GWAS, including unrelated European descent individuals from UK Biobank (UKB, *N* = 386,533, 46.0% female) and 23andMe (*N* = 944,477, 53.1% female) (Jansen et al., 2019). Insomnia cases were measured with questionnaire data and defined as participants who usually have trouble in falling asleep at night or wake up in the middle of the night in the UKB cohort, and were diagnosed with participants affirming no less than one phenotypic concept concerning inferior sleep status in the 23andMe cohort. The prevalence of insomnia was 29.9% in the combined sample of UKB and 23andMe and was higher in females (34.6%) than males (24.5%).

Genetic association estimates with sleep duration were obtained from the UKB participants of European ancestry (*N* = 446,118, 54.1% female) (Dashti et al., 2019). The GWAS examined the following three sleep duration phenotypes: self-reported habitual sleep duration (continuous variable), which was assessed by the question: “About how many hours sleep do you get in every 24 h? (please include naps).” The answer was responded in hour increments and could only contain integer values; short and long sleep duration (binary variable), categorized as <7 h and >8 h relative to 7–8 h sleep duration, respectively. The mean sleep duration was 7.2 h (1.1 standard deviation) per day, and the prevalence for short and long sleep duration ware 25.8 and 10.1%, respectively.

Full summary statistics for morningness were acquired from the largest meta-analysis of GWAS among adults of European ancestry, including 449,734 participants from UKB and 248,098 participants from 23andMe (Jones et al., 2019). The participants in the UKB cohort were promoted to answer the question “Do you consider yourself to be?” with six possible answers, persons answering “Definitely a ‘morning’ person” or “More a ‘morning’ than ‘evening’ person” were assigned to cases of morningness, and persons answering “More an ‘evening’ than a ‘morning’ person” or “Definitely an ‘evening’ person” were assigned to controls. The participants in the 23andMe cohort responded to the question “Are you naturally a night person or a morning person?” with two possible answers. 57.2% of the individuals were coded as a morning person in the pooled cohort, and the percentages were 62.6% and 48.6% for UKB and 23andMe, respectively.

GWASs for T2DM were selected to extract genetic association information for the outcome. When using genetic consortia that have significant overlapping sets in the exposure and outcome GWAS, the two-sample summary data-based MR may develop biased estimates (Burgess et al., 2016). Thus, we excluded the GWASs that involving UKB or 23andMe as main or sub cohort. Furthermore, to reduce the possible confounding derived from population stratification, we restricted the T2DM cohort to European-descent adults. According to the above criteria, we drew on summary statistics from the largest GWAS of T2DM, which was conducted by the DIAbetes Genetics Replication And Meta-analysis (DIAGRAM) consortium and contained 18 study cohorts and a total of 159,208 participants (Scott et al., 2017). The T2DM diagnosis criteria, control selection principles, and study characteristics for each cohort had been described in more detail in the original article. To examine the solidity of the findings, we also selected another newly released T2DM GWAS, which was derived from FinnGen cohort and contained a total of 131,616 individuals, as the validation sample.

### Selection of Instruments

The first core assumption for MR is that the instruments are robustly and strongly associated with the exposure of interest. When the relationship of the instruments with exposure is weak, the causal estimate will be biased toward the null, which is referred to as weak instrument bias (Davies et al., 2015). To address the potential weak instrument bias, we included the independent lead single nucleotide polymorphisms (SNPs) that are genome-wide significantly (*P* < 5 × 10^–8^, *r*^2^ < 0.1) associated with the sleep traits as preliminary instruments. Then we extracted the summary statistics for the associations of the selected instruments with T2DM from the T2DM GWAS database and matched the two groups of sample data based on the SNP ID. To make sure that the effect of an instrument on the exposure and the effect of that instrument on the outcome each correspond to the same allele, we performed harmonization of the direction of effects (Hemani et al., 2018). To further ensure the independence of the instruments, SNPs that were in linkage disequilibrium (LD) were excluded from the instrument variable set using the clumping algorithm (r^2^ threshold = 0.01 and window size = 1 Mb) (Chang et al., 2015).

### Investigation of Pleiotropy

Other main identifying assumptions for an MR analysis are that the instrument is not associated with the confounding factors, and it influences the outcome only through the exposure. These two assumptions can be together summarized as independent of pleiotropy (Emdin et al., 2017). Pleiotropy occurs when the genetic variant influences the target outcome via any pathway other than the exposure. The nature of pleiotropy could invalidate an instrument, thus bias the estimate in the MR analysis (Zhu et al., 2018). Pleiotropy is commonplace in practice, however, a complete understanding of the effect of genetic variants on the phenotypes is lacking. Therefore, it is necessary to take full advantage of statistical findings to assess and identify the potential pleiotropic instruments (Swerdlow et al., 2016). We first investigated the association of the instruments with major confounders such as body mass index, alcohol use and physical activity, and excluded the SNPs that were associated with these known confounders at the genome-wide significance level (Chen et al., 2011; Van Reen et al., 2011; Bayon et al., 2014; Semplonius and Willoughby, 2018). Known potentially pleiotropic effects of the chosen SNPs were obtained with PhenoScanner, a database that provides massive human genotype-phenotype associations (Staley et al., 2016). We then performed the individual Cochran’s Q outlier test to detect the unknown pleiotropic effects of the instruments (Del Greco et al., 2015). The ratio estimate (i.e., the estimate for the SNP–outcome association divided by the estimate for the SNP-exposure association) of each valid instrument will only vary by chance, and a significant heterogeneity would hint at the violation of assumptions in the instrument, most likely as a result of pleiotropy. These outlier SNPs with significant contributions to the Q statistic for heterogeneity were removed from the instrument variable set.

After the above steps to eliminate the potential pleiotropy, global Q test was implemented to examine if there is still heterogeneity among the screened instruments, and MR-Egger regression was also conducted to evaluate the directional pleiotropy of the instruments (Bowden et al., 2015). Directional pleiotropy here refers to the pleiotropic effects of genetic variants are not balanced about the null. In this situation, the estimations from MR analyses inevitably suffer from bias (Burgess and Thompson, 2017). A significant deviation of the MR-Egger intercept from 0 indicated directional pleiotropy in the instruments.

### Main MR Analysis

After a series of examinations for the validity of the instruments, we evaluated the causal estimations with the inverse-variance weighted (IVW) method, which essentially models the weighted regression of SNP-outcome effects on SNP-exposure effects where the intercept is constrained to zero (Burgess et al., 2013). The modified weights in the IVW framework take account of the uncertainty of SNP–exposure associations and move beyond the “NO Measurement Error” (NOME) assumption, therefore leveraging more power compared with the IVW using the first-order weights (Bowden et al., 2019). Given this, we adopted the modified IVW approach to obtain the estimates for the causal effect of the sleep traits on T2DM.

### Sensitivity Analyses and Other Elements

To assess the extent to which findings were robust to potential pleiotropy, we also performed sensitivity analysis with four other established MR methods: IVW with first-order weights (Burgess et al., 2013), simple weighted median (SME), weighted median estimates (WME) (Bowden et al., 2016), and MR-Egger (Bowden et al., 2015). IVW with first-order weights could produce consistent estimates when there are no pleiotropic instruments, whereas the median based and MR-Egger estimates allow the inclusion of the pleiotropic instruments and are relatively robust to pleiotropy, although at the cost of reduced statistical power. Consistent estimates across multiple methods strengthen the robustness of the causal findings. Furthermore, we conducted the analyses by combining the five major sleep traits with both the main DIAGRAM T2DM cohort and the alternative FinnGen T2DM cohort respectively, to assess the consistency of the results. Lastly, we exploited the three largest GWAS cohorts from the MAGIC consortium and investigated the causal relationships of five sleep-related traits with Hemoglobin A1c(Hb1Ac), fasting glucose (FG), and fasting insulin (FI).

To account for multiple testing, we employed a Bonferroni-corrected threshold of *P* < 0.005 (0.05/10 to correct for five sleep traits in relation to two T2DM outcomes). A *P*-value between 0.005 and 0.05 was considered as suggestive evidence of causality and needs to be further confirmed. The statistical analyses were performed in two-tailed, with the use of TwoSampleMR package (perform data extraction, harmonization, and clumping), RadialMR package (perform modified IVW and Q test), and MendelianRandomization package (query the genotype-phenotype associations, perform sensitivity analysis and MR-Egger intercept test) in R project 3.5.0.

## Results

### Insomnia and T2DM

**FIGURE 1:**
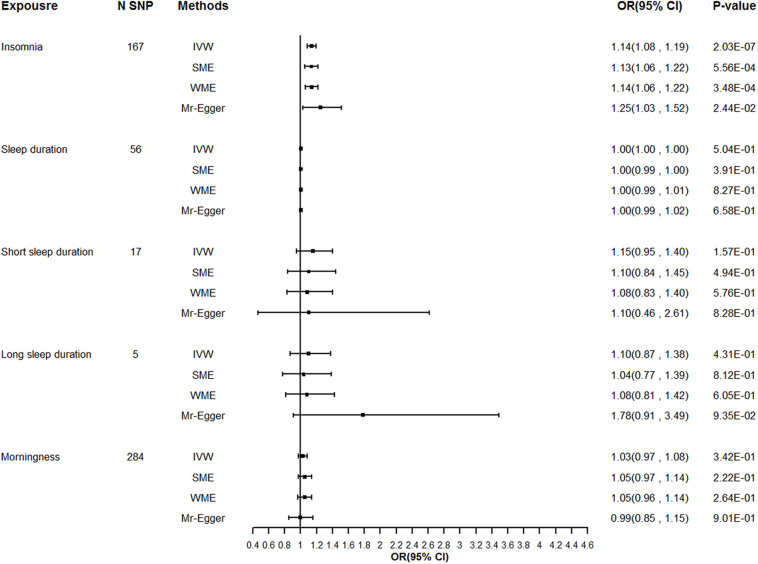
Sensitivity analysis for the causality of sleep traits with the risk of T2DM. T2DM, Type 2 diabetes mellitus; N SNPs, number of SNPs retained and used in the MR analysis after filtered by individual Q outlier test; OR, odds ratio; CI, confidence interval.

We extracted summary association statistics for the 248 genome-wide significant SNPs previously demonstrated to be associated with insomnia. We then matched and harmonized the effects for the SNPs on insomnia and on T2DM to each be for the same reference allele. Thirty-seven SNPs were excluded because of high LD with the other SNPs, 22 SNPs were excluded due to their significant relationships with the known confounders, and 22 outliers were identified by the individual Q test and were removed from the instrument variable set. Global Q test (*Q* = 138.04, *P* = 0.94) and MR-Egger test (intercept *P* = 0.32) did not support any evidence for heterogeneity or directional pleiotropy for the rest of the instruments. Detailed information on the instruments for insomnia was shown in Supplementary Table 1 and Supplementary Figure 1. Modified IVW supported the findings of a significant adverse effect of insomnia on the risk of T2DM [odds ratio (OR) = 1.14, 95% confidence interval (CI): 1.09–1.19, *P* = 1.29E–08], and the estimates were broadly consistent between the main analysis and sensitivity analysis (Table 2 and Figure 1).

**TABLE 2:** Main MR analysis for the causality of sleep traits with the risk of T2DM.

Phenotypes		MR results		
Exposure	Outcome	N SNPs	OR (95% CI)	*P*-value
Insomnia	T2DM	167	1.14 (1.09, 1.19)	1.29E–08
Sleep duration		56	1.00 (1.00, 1.00)	0.43
Short sleep duration		17	1.15 (0.96, 1.38)	0.09
Long sleep duration		5	1.10 (0.79, 1.51)	0.43
Morningness		284	1.03 (0.98, 1.08)	0.29

*MR, Mendelian randomization; T2DM, Type 2 diabetes mellitus; N SNPs, number of SNPs retained and used in the MR analysis after filtered by individual Q outlier test; OR, odds ratio; CI, confidence interval.*

Since a significant effect of insomnia on T2DM was observed, we further conducted a complimentary analysis to assess the causal effect of T2DM on the risk of insomnia. Each genome-wide significant T2DM-related instrument and its association estimates with T2DM and insomnia are presented in Supplementary Table 2 and Supplementary Figure 2. However, across all MR methods, we found no evidence of the causal relationship of T2DM with insomnia (Supplementary Table 3).

### Sleep Duration and T2DM

After harmonization of the SNP effects in the two summary datasets (UKB for sleep duration and DIAGRAM for T2DM), there were 77, 27, and 6 SNPs used to instrument sleep duration, short sleep duration, and long sleep duration. After clumping for the selected SNPs, we removed rs7115226 and rs142180737 from the sleep duration and short sleep duration instrument set, respectively, due to their LD with other SNPs. We then queried the left SNPs for their associations with the phenotypes and filtered 10, 4, and 1 SNPs which were significantly correlated with the given confounders. Subsequently, the individual Q test detected 10 outliers and 5 outliers from the sleep duration and short sleep duration instrument set, respectively. The removal of these outliers resulting in a final number of 56, 17, and 5 SNPs that acted as instruments for sheep duration, short sleep duration, and long sleep duration, respectively. Evidence for heterogeneity provided by global Q test did not indicate any violation of the MR assumptions (*Q* = 39.70, *P* = 0.94 for sleep duration; *Q* = 11.35, *P* = 0.79 for short sleep duration; *Q* = 4.37, *P* = 0.36 for long sleep duration), and the directional pleiotropy estimated by MR-Egger test was consistent with the null for all of the models (intercept *P* = 0.51 for sleep duration; intercept *P* = 0.92 for short sleep duration; intercept *P* = 0.14 for long sleep duration). Resulting lists of instrument SNPs are given in Supplementary Tables 4–6 and Supplementary Figures 3–5.

Little evidence for a causal effect of sleep duration on T2DM was observed either with the modified IVW (OR 1.00, 95% CI: 1.00–1.00, *P* = 0.43), first-order IVW or the pleiotropy robust methods (simple median, weighted median, MR-Egger) applied. Similarly, we found no causal relationship of either short sleep duration (OR 1.15, 95% CI: 0.96–1.38, *P* = 0.09) or long sleep duration with T2DM in the primary analysis (OR 1.10, 95% CI: 0.79–1.51, *P* = 0.43), and the sensitivity analysis yielded a similar pattern of results (Table 2 and Figure 1).

### Morningness and T2DM

We implemented MR analysis using 343 SNPs that are strongly associated with morningness as instruments. Among the instruments, 6 SNPs were ruled out after the clumping process, 19 SNPs were significantly associated with the confounders thus been excluded from the instrument set, and 34 potential outlier SNPs were picked out with the individual Q test. When restricting the MR model to the remaining 284 instruments, the global Q statistic indicated no notable heterogeneity (*Q* = 228.37; *P* = 0.99), and the MR-Egger test also suggested no horizontal pleiotropy (intercept *P* = 0.62). Details of the selected instruments are provided in Supplementary Table 7 and Supplementary Figure 6. No significant causal relationship of genetically determined morningness with T2DM was suggested with the modified IVW (OR 1.03, 95% CI: 0.98–1.08, *P* = 0.29), and the effect estimates with other sensitivity analyses methods were largely unchanged compared to the main analysis (Table 2 and Figure 1).

### Other Analyses

We acquired quite similar results when replicating the causal estimations using the FinnGen T2DM cohort. After excluding the correlated and pleiotropic SNPs, the valid instruments for insomnia exhibited a significant effect on the risk of T2DM. Furthermore, we found suggestive evidence for the effect of sleep duration and morningness on T2DM risk with the modified IVW model, whereas the nominally significant effects disappeared when utilizing alternative MR models in the sensitivity analyses. One possible reason for the inconsistency is that the modified IVW method accounts for the sampling errors in the estimated effect sizes of the instruments on the exposure, therefore it is more powerful than other MR methods. Meanwhile, the pleiotropy-corrected approaches such as median-based estimations and MR-Egger regression introduce noise to the causal association, which means that the statistical power will be reduced (Bowden et al., 2019; Dudbridge, 2020). Since the causation of sleep duration and morningness on T2DM failed to reach the Bonferroni-adjusted significance, the suggestive evidence for the relationships should be investigated further (Supplementary Figure 7). Also, we acquired a similar pattern of results for the causality of sleep with Hb1Ac, FG, FI (Supplementary Figure 8).

## Discussion

The biological mechanisms of the relationship between sleep and diabetes are complicated, sleep habits and sleep disturbance, such as sleep duration, insomnia, and different kinds of circadian rhythms may act on T2DM through different biological mediations. Defining the relationship between these sleep traits and glycemic health is of great importance in understanding the detailed mechanisms and discovering potential treatment strategies for T2DM disease (Anothaisintawee et al., 2016; Ding et al., 2019). Therefore, we investigated the causality of different sleep traits with T2DM in this study and found significant evidence for an adverse effect of insomnia on T2DM risk. However, no evidence of causal association was found in other sleep-related phenotypes with T2DM. The study enhances the understanding of T2DM and opens new potential avenues for T2DM intervention and therapy, thus making a positive endeavor on public health and medical care.

Previous epidemiological studies provide inconsistent results in terms of the relationship between sleep and T2DM (Yaggi et al., 2006; Chaput et al., 2007; Shan et al., 2015). A cohort study observed that both short or long sleep duration increase the risk of developing T2DM independently (Chaput et al., 2007). While another study found that there is no significant association between sleep duration and a higher risk of T2DM after adjusting for possible factors (Hayashino et al., 2007). The mixed results can be attributed partly to the existence of unmeasured confounders and reverse causation, which has distorted effect estimates of the observational studies. However, when the studies are designed reasonably and the core assumptions are tested rigorously, valid causal evidence can always be achieved through the MR approaches. Considering this, we leverage a series of MR methods to investigate the causal relation of sleep with T2DM and acquire credible results.

Recent studies have offered some supporting evidence for the causal link between insomnia and T2DM. A longitudinal observation study demonstrated that insomnia patients were more likely to develop T2DM than the comparison cohort at about 16% higher. Furtherly, with an increased duration of insomnia symptoms, the risk of T2DM also tended to increase (Lin et al., 2018). Besides, an experimental study induced sleep deprivation in healthy individuals and found that insomnia led to hyperglycemia and insulin resistance, which was reversed subsequently when their sleep returned to normal (Benedict et al., 2011; Rao et al., 2015). Moreover, a large retrospective cohort study including more than 80,000 pre-diabetic people indicated that after adjusting for traditional risk factors, people who suffer from insomnia were 28% more likely to develop T2DM than those without insomnia symptoms (LeBlanc et al., 2018). The external evidence strengthens our confidence in the generalizability and validity of the present findings. All of these findings, including our study, have provided ample and credible evidence for a causal effect of insomnia on T2DM.

Furtherly, several potential mechanisms may contribute to the causal relationship between insomnia and T2DM. A previous study showed that insomnia may promote activation of the sympathetic nerve thereby increasing insulin resistance, which plays an important role in the risk of developing T2DM (Irwin et al., 2003). Furthermore, insomnia is also associated with the activation of chronic systemic inflammation, leading to the presence of insulin resistance which eventually develops into T2DM (Wang et al., 2013; Irwin et al., 2016). Despite these findings, further work to uncover the in-depth causal mechanisms is required.

It has long been uncertain whether the association between insomnia and T2DM is owed potentially to a negative causal relationship of insomnia on T2DM, and/or a relationship between T2DM and more serious insomnia symptoms. Some studies have reported that about half of the participants with T2DM also suffered from insomnia, indicating that insomnia itself is a further complication of T2DM (Luyster and Dunbar-Jacob, 2011; Koopman et al., 2020). To better understand the direction of the relationship, we also evaluated the causal effect of T2DM on insomnia but found non-significant results. The potential reason for the observed T2DM-insomnia relationship maybe that observational studies cannot control for all confounding factors like physical activities, which related both sleep and T2DM and thus induce a major bias for the estimation (Semplonius and Willoughby, 2018).

This study has important strengths. First, this study explored the causality between a broad range of sleep-related traits and T2DM, contributing to filling the gaps over the existing observational studies and extending the relevant research notably. Second, we exploited the MR design and analysis to control for reverse causality and unmeasured confounding, which might lead to biased results in the traditional observational studies. Third, we took a series of steps to make sure the MR core assumptions are satisfied and the estimates are valid. Specifically, the instruments of sleep-related phonotypes came from large scale GWASs, which provided strongly and robustly associated SNPs and averted the potential weak instrument bias. Furthermore, the pleiotropic SNPs were identified and the validity of the reserved instruments was examined through different tests to correct for the bias deriving from pleiotropy. Fourth, the replication study for the relationship of sleep with another T2DM cohort ensured the solidity of the results. Last, the analyses included a large number of sample sizes and SNPs leveraging from GWASs, thereby offering sufficient statistical power for the causal estimation. These measures together help increase confidence in the results.

Nevertheless, our study has several limitations. First, we could not investigate the non-linear effects of sleep traits on T2DM due to the summary statistics we used, resulting in hardly assessable U-shaped associations. Second, the sleep-related data were obtained from self-reported questionnaires surveys, some of which may be less exact than directly objective measurements, such as sleep duration. Although previous studies have proved the validity of subjective sleep, people often overestimated their sleep duration time by up to 1 h, which may lead to imprecise results (Lockley et al., 1999). Further work can attempt to use the device-measured sleep duration to evaluate its association with T2DM. Moreover, recent studies indicated that there are gender or age differences in people with T2DM, but we could not investigate these differences due to the limitation of a lack of data (Lai et al., 2013).

Overall, we concluded from this study that there is strong evidence for a causal effect of insomnia on T2DM risk. The potentially modifiable sleep traits should be added to the prevention strategies of T2DM to improve public health. Moreover, this study highlights the need for further research regarding the mechanisms underlying these causal associations and leads to optimized medical care and management of T2DM.

## Data Availability Statement

The original contributions presented in the study are publicly available. The data can be found at GWAS Catalog (https://www.ebi.ac.uk/gwas/), DIAGRAM (http://diagram-consortium.org/index.html), FinnGen (https://finngen.gitbook.io/documentation/), and MAGIC (https://www.magicinvestigators.org/).

## Author Contributions

TW, XG, and HS conceived and designed the whole study. YZ acquired and interpreted the data. XG, JW, and LL conducted the statistical analysis. XG and HS drafted the initial manuscript. TW supervised the whole study and attested to the integrity of the data and the accuracy of the data analysis. All authors reviewed and revised the manuscript, and approved the final version.

## Conflict of Interest

The authors declare that the research was conducted in the absence of any commercial or financial relationships that could be construed as a potential conflict of interest.
