# Supplementary material for: Investigating Causal Relations Between Sleep-Related Traits and Risk of Type 2 Diabetes Mellitus: A Mendelian Randomization Study
Source: Front Genet. 2020 Dec 15;11:607865. doi: 10.3389/fgene.2020.607865 (PMC7770175; doi:10.3389/fgene.2020.607865)
Supplement: Supplementary file 1 [file Image_1.PDF]

Supplementary Figures 1-8

Supplementary Figure 1: Scatter plot of SNP-insomnia and SNP-T2DM association with overlay of causal estimate from IVW test in two-sample MR-analysis

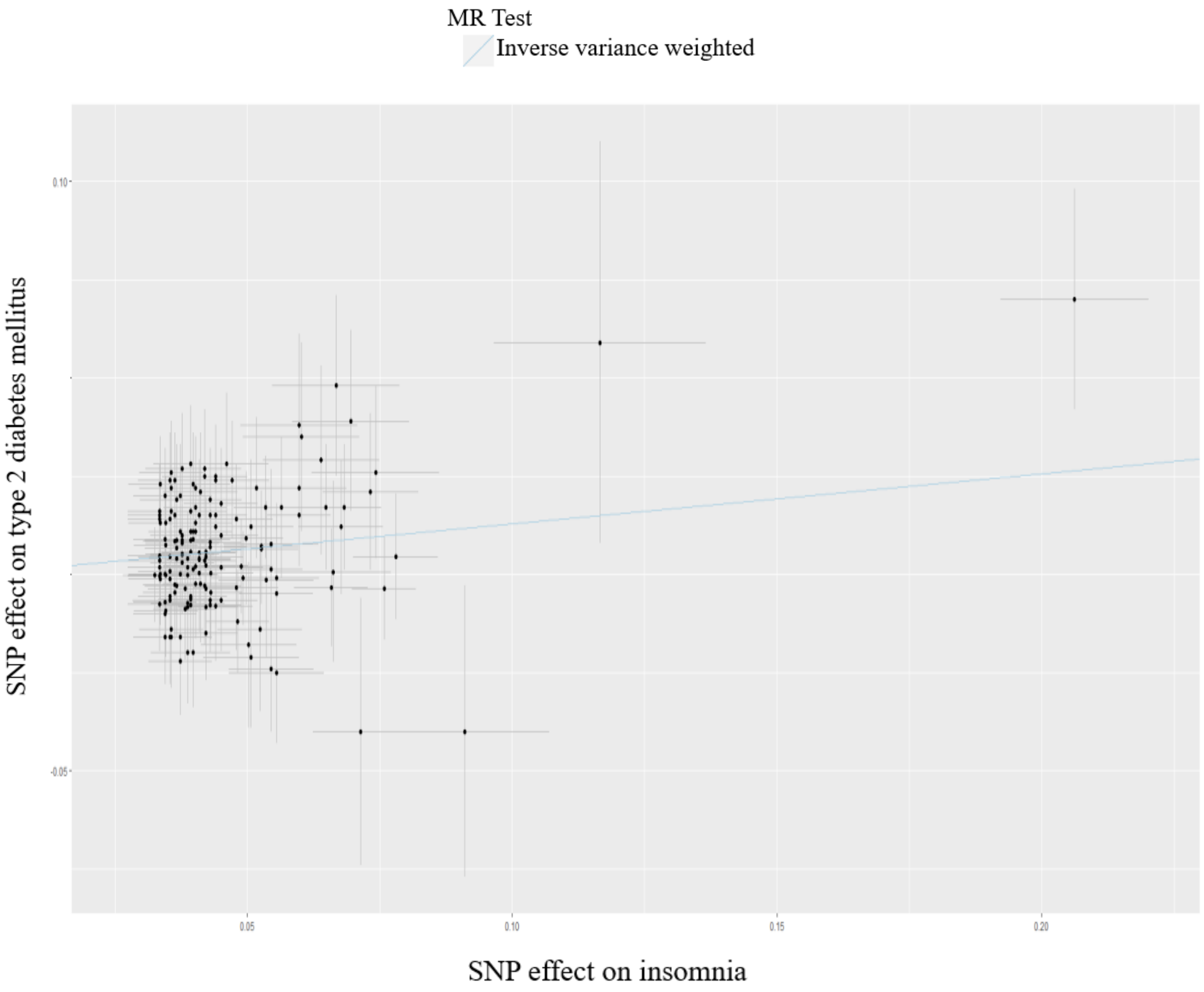

Supplementary Figure 2: Scatter plot of SNP-T2DM and SNP-insomnia association with overlay of causal estimate from IVW test in two-sample MR-analysis

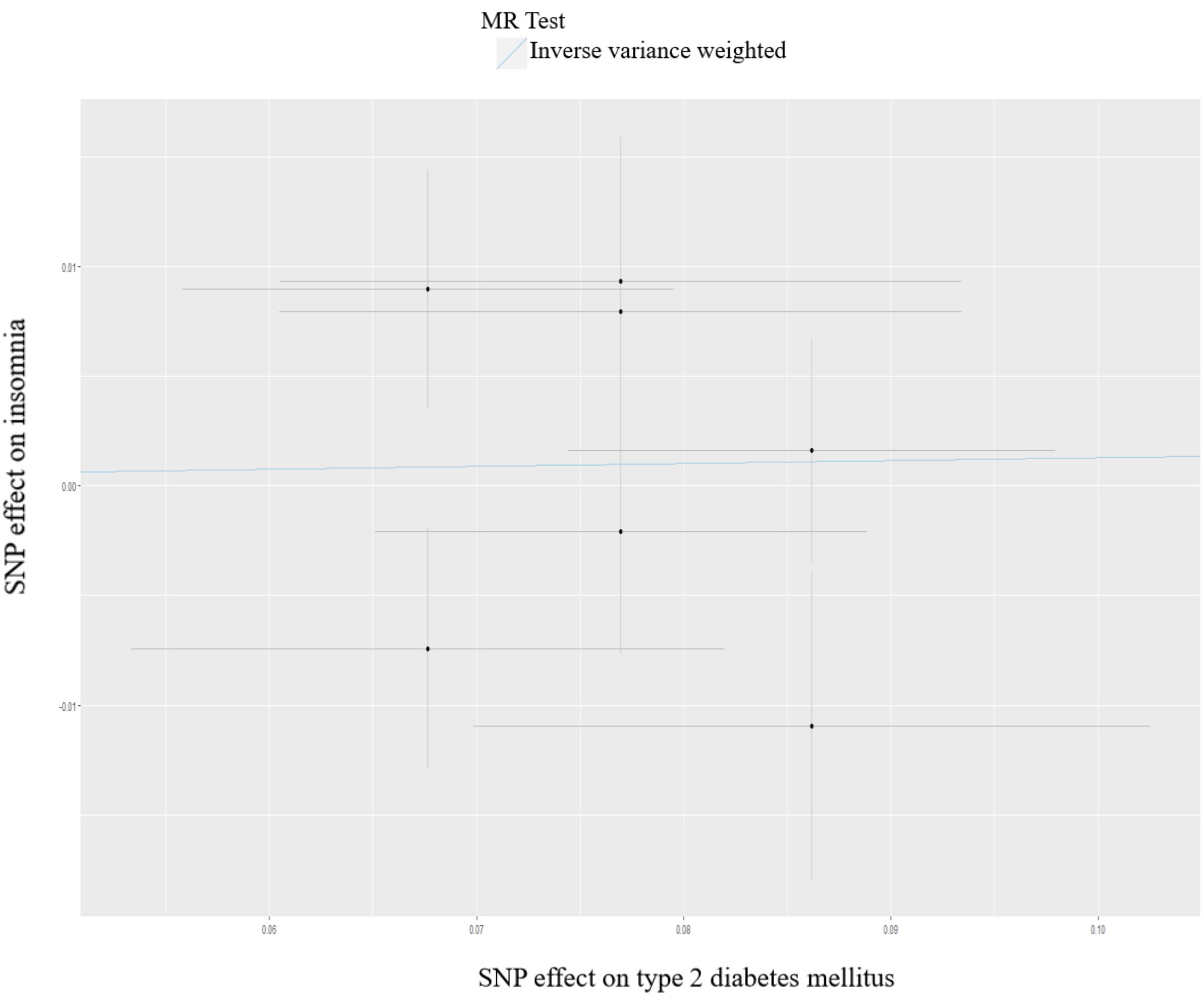

Supplementary Figure 3: Scatter plot of SNP-sleep duration and SNP-T2DM association with overlay of causal estimate from IVW test in two-sample MR-analysis

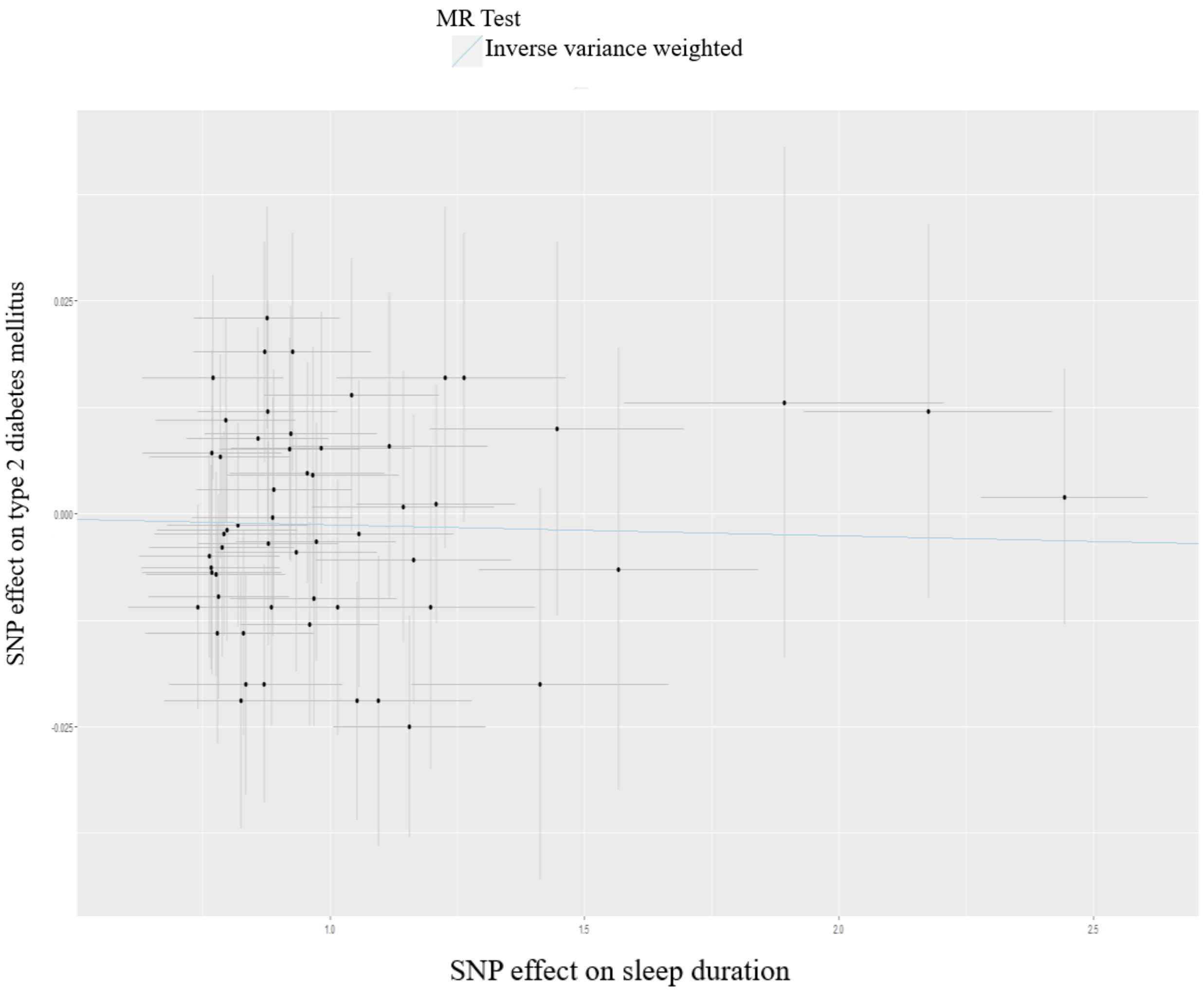

Supplementary Figure 4: Scatter plot of SNP-short sleep duration and SNP-T2DM association with overlay of causal estimate from IVW test in two-sample MR-analysis

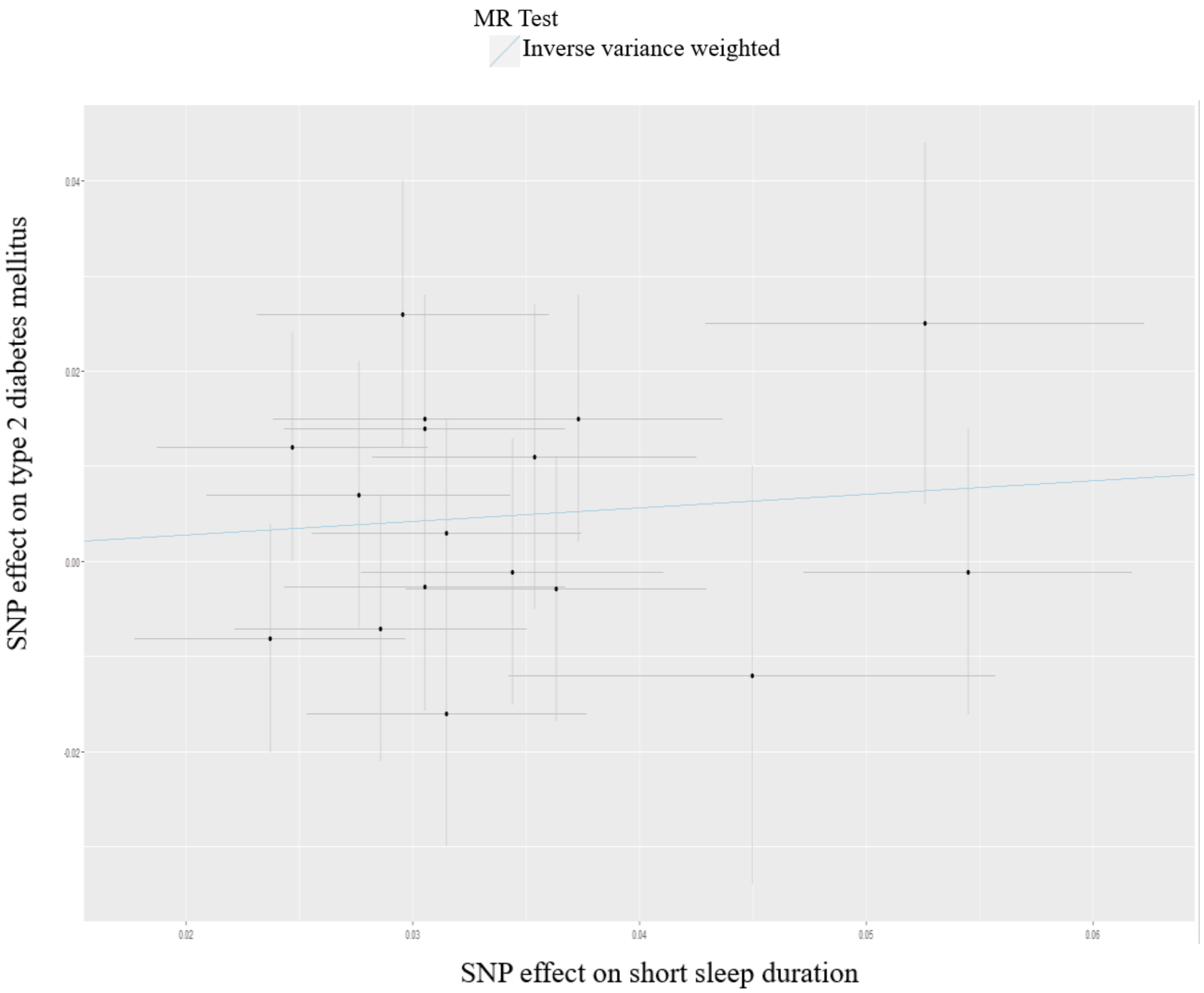

Supplementary Figure 5: Scatter plot of SNP-long sleep duration and SNP-T2DM association with overlay of causal estimate from IVW test in two-sample MR-analysis

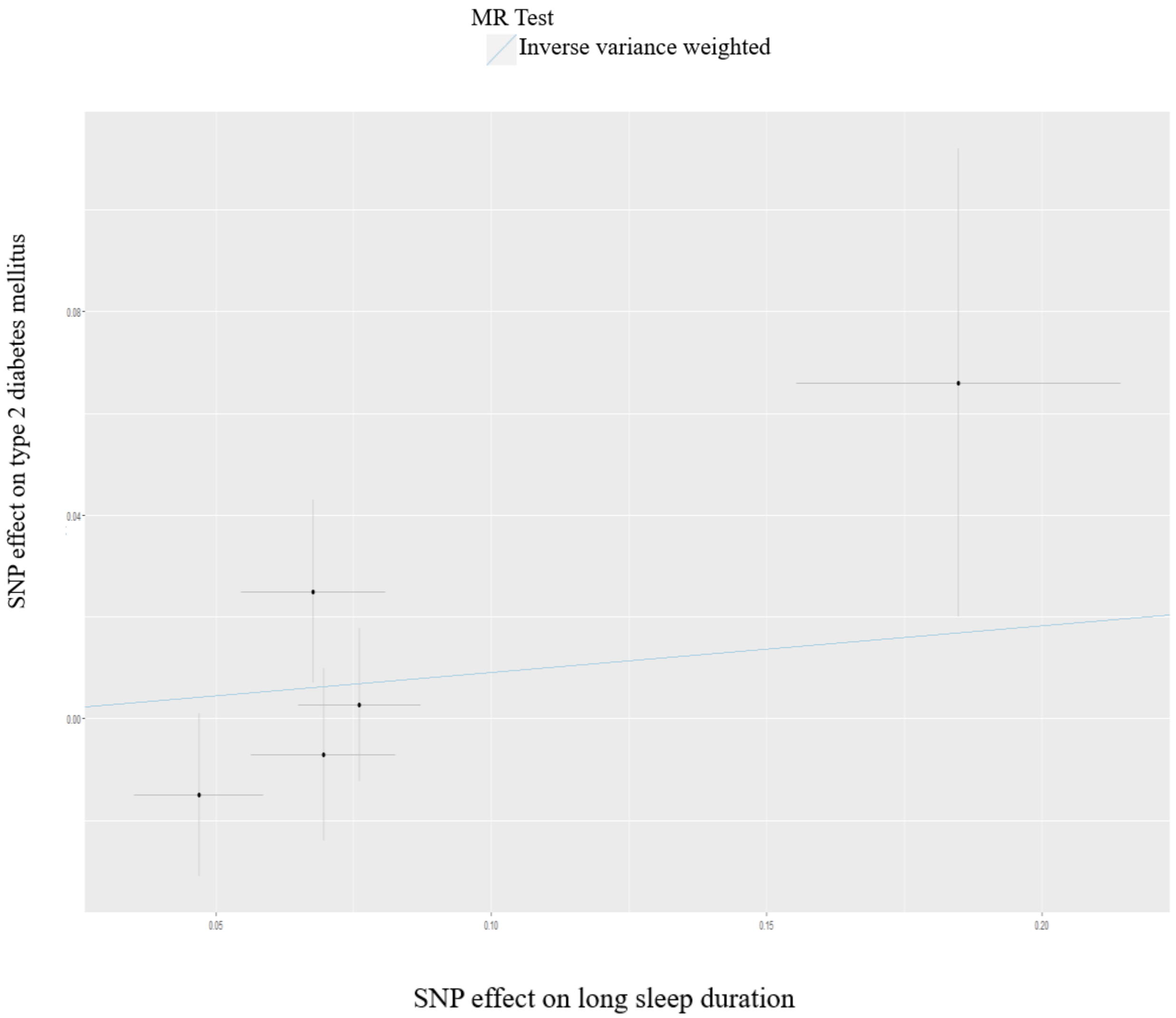

Supplementary Figure 6: Scatter plot of SNP-morningness and SNP-T2DM association with overlay of causal estimate from IVW test in two-sample MR-analysis

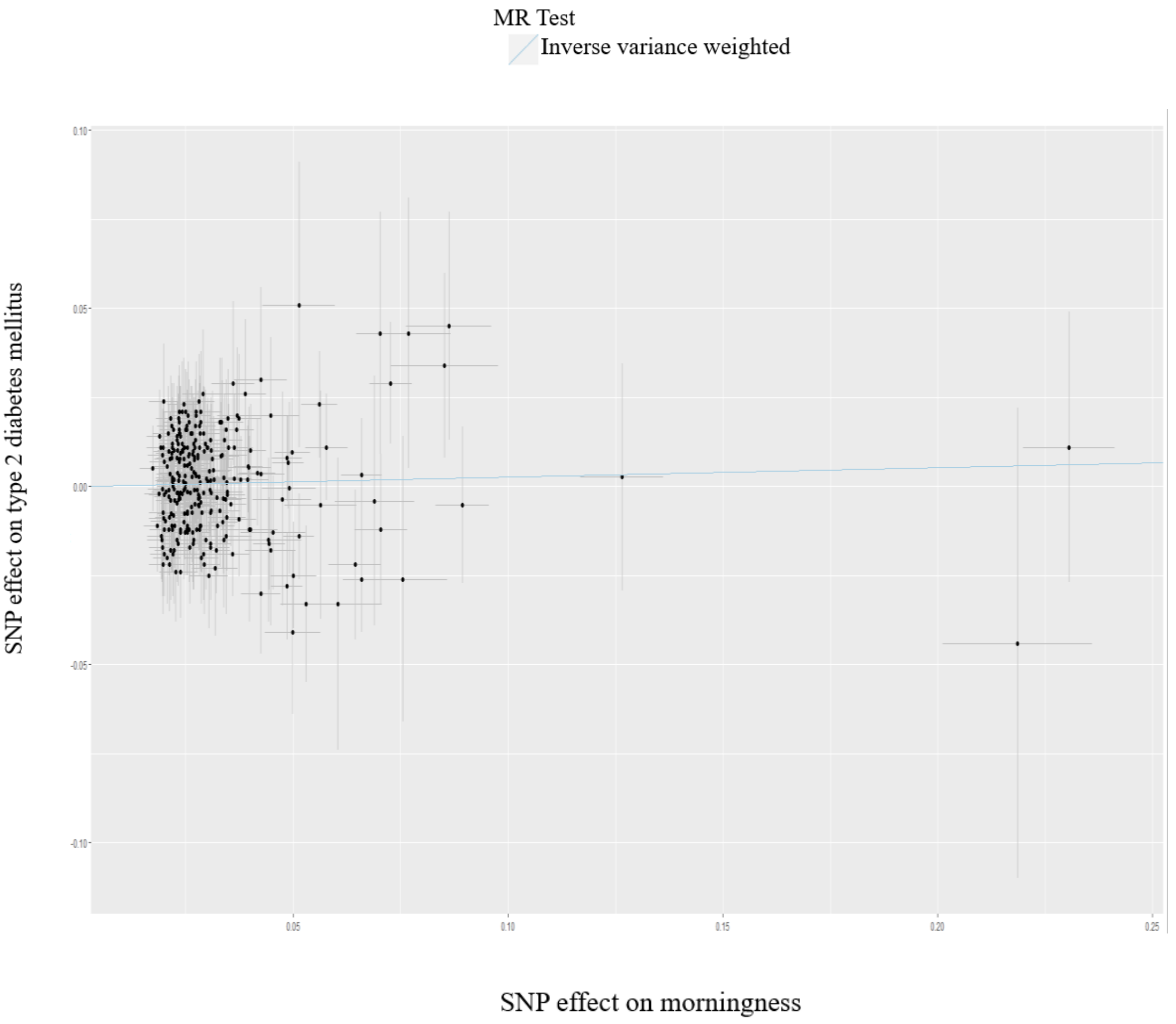

Supplementary Figure 7: MR causal relationship of sleep traits with T2DM (FinnGen cohort)

| Expousre | N SNP | Methods | OR(95% CI) | P-value |
|---|---|---|---|---|
| Insomnia | 162 | Radial_IVW | 1.11(1.06 , 1.17) | 3.18E-05 |
| | | IVW | 1.11(1.06 , 1.18) | 9.67E-05 |
| | | SME | 1.09(1.01 , 1.18) | 2.71E-02 |
| | | WME | 1.09(1.01 , 1.19) | 3.23E-02 |
| | | Mr-Egger | 1.14(0.93 , 1.40) | 2.02E-01 |
| Sleep duration | 54 | Radial_IVW | 1.00(1.00 , 1.01) | 2.72E-02 |
| | | IVW | 1.00(1.00 , 1.01) | 4.72E-02 |
| | | SME | 1.00(1.00 , 1.01) | 1.99E-01 |
| | | WME | 1.00(1.00, 1.01) | 2.18E-01 |
| | | Mr-Egger | 0.99(0.98 , 1.01) | 3.93E-01 |
| Short sleep duration | 18 | Radial_IVW | 0.92(0.78 , 1.09) | 3.03E-01 |
| | | IVW | 0.92(0.74 , 1.15) | 4.76E-01 |
| | | SME | 0.83(0.62 , 1.12) | 2.30E-01 |
| | | WME | 0.94(0.70 , 1.26) | 6.66E-01 |
| | | Mr-Egger | 1.74(0.67 , 4.51) | 2.57E-01 |
| Long sleep duration | 5 | Radial_IVW | 1.03(0.77 , 1.38) | 7.70E-01 |
| | | IVW | 1.03(0.81 , 1.31) | 8.04E-01 |
| | | SME | 1.09(0.78 , 1.54) | 6.09E-01 |
| | | WME | 1.05(0.77 , 1.43) | 7.50E-01 |
| | | Mr-Egger | 1.41(0.68 , 2.95) | 3.58E-01 |
| Morningness | 269 | Radial_IVW | 0.94(0.89 , 1.00) | 4.64E-02 |
| | | IVW | 0.94(0.89 , 1.00) | 6.77E-02 |
| | | SME | 0.96(0.87 , 1.05) | 3.44E-01 |
| | | WME | 0.95(0.87 , 1.05) | 3.14E-01 |
| | | Mr-Egger | 0.90(0.75 , 1.07) | 2.17E-01 |

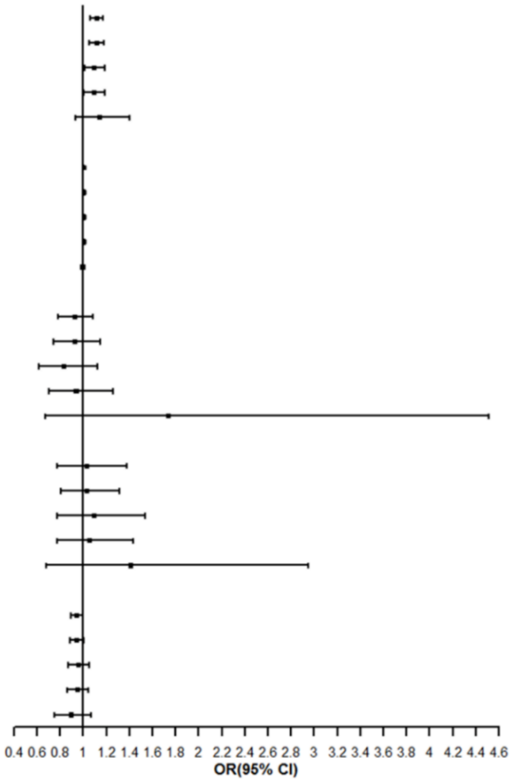


Abbreviations: MR: Mendelian randomization; T2DM: Type 2 diabetes mellitus; N SNPs: number of SNPs retained and used in the MR analysis after clumping and exclusion of pleiotropic SNPs; OR: odds ratio; CI: confidence interval.

Supplementary Figure 8: MR causal relationship of sleep traits with HbA1c, FG and FI

| Expousre | Outcome | N SNPs | Beta(95% CI) | P-value |
|---|---|---|---|---|
| **MR causal relationship of sleep traits with HbA1c (N=123,665)** | | | | |
| Insomnia | | 94 | 0.014(0.005,0.023) | 1.91E-03 |
| Sleep duration | | 19 | 0.000(-0.001,0.001) | 0.724 |
| Short sleep duration | HbA1c | 13 | 0.017(-0.027,0.061) | 0.393 |
| Long sleep duration | | 1 | -0.009(-0.084,0.066) | 0.817 |
| Morningness | | 133 | -0.004(-0.015,0.007) | 0.449 |
| **MR causal relationship of sleep traits with FG (N=140,595)** | | | | |
| Insomnia | | 159 | 0.023(0.013,0.034) | 9.58E-06 |
| Sleep duration | | 52 | 0.000(-0.001,0.001) | 0.881 |
| Short sleep duration | FG | 20 | 0.003(-0.041,0.048) | 0.874 |
| Long sleep duration | | 4 | -0.039(-0.128,0.049) | 0.385 |
| Morningness | | 258 | -0.007(-0.02,0.006) | 0.289 |
| **MR causal relationship of sleep traits with FI (N=98,210)** | | | | |
| Insomnia | | 157 | 0.026(0.013,0.039) | 7.64E-05 |
| Sleep duration | | 52 | 0.001(0.000,0.002) | 0.248 |
| Short sleep duration | FI | 21 | -0.008(-0.043,0.028) | 0.647 |
| Long sleep duration | | 4 | -0.030(-0.110,0.051) | 0.470 |
| Morningness | | 257 | -0.022(-0.037,-0.008) | 0.003 |

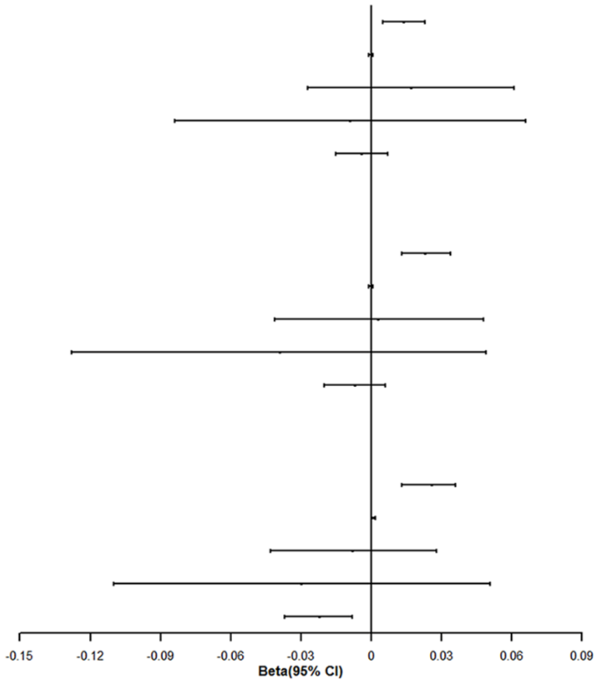


Abbreviations: MR: Mendelian randomization; HbA1c: Hemoglobin A1c; FG: fasting glucose; FI: fasting insulin; N SNPs: number of SNPs retained and used in the MR analysis after clumping and exclusion of pleiotropic SNPs; OR: odds ratio; CI: confidence interval.

Note: the causality of long sleep duration with T2DM are estimated with classical IVW due to the lack of instruments, other causalities were estimated with modified IVW.
